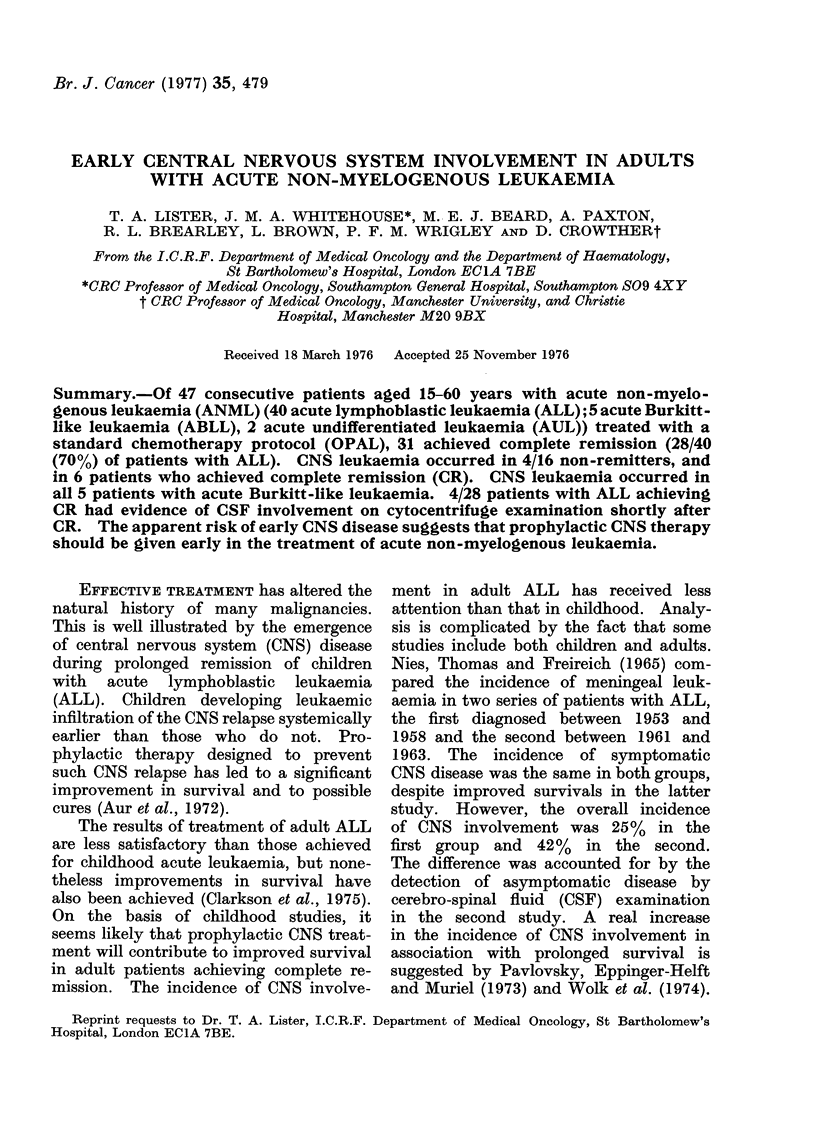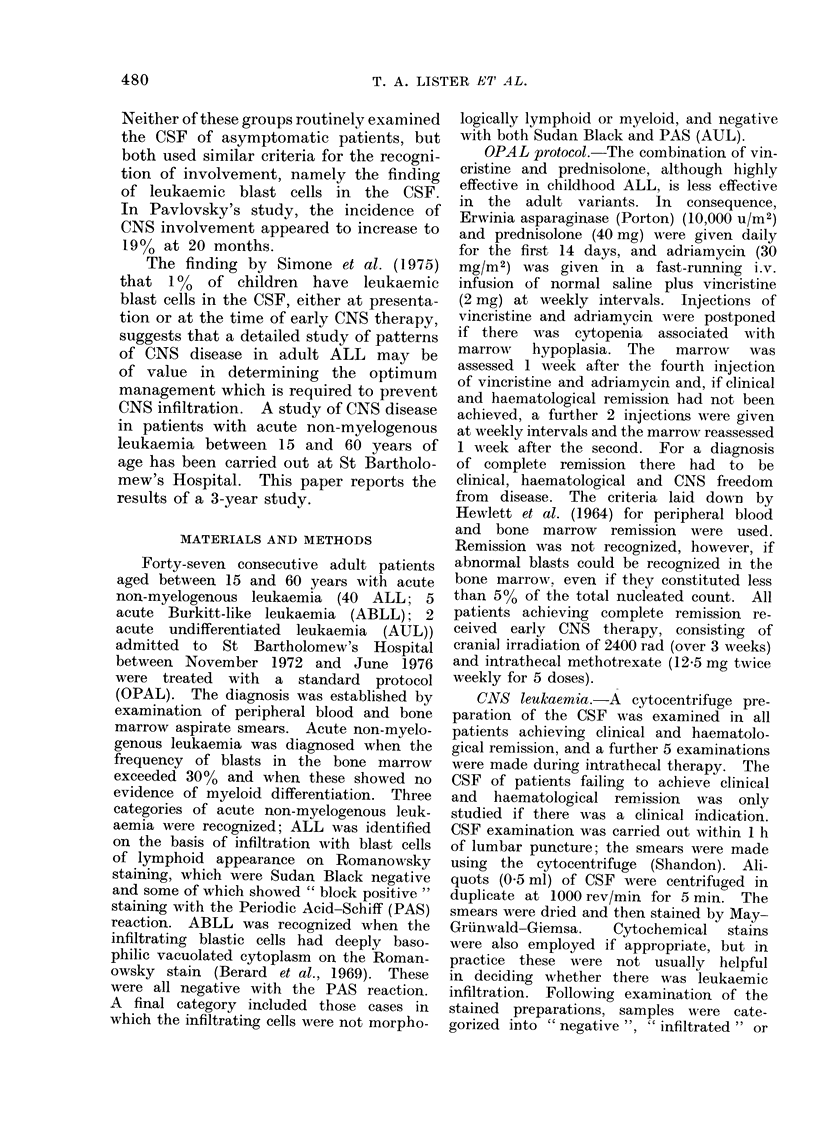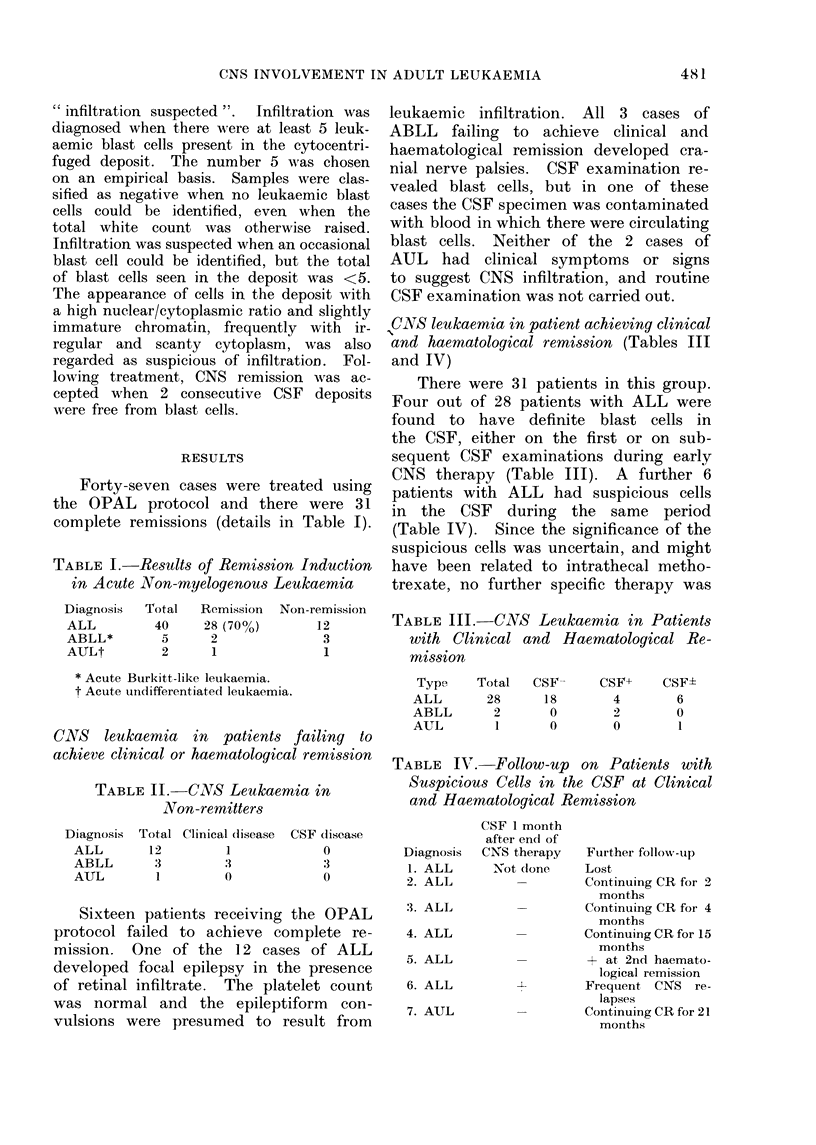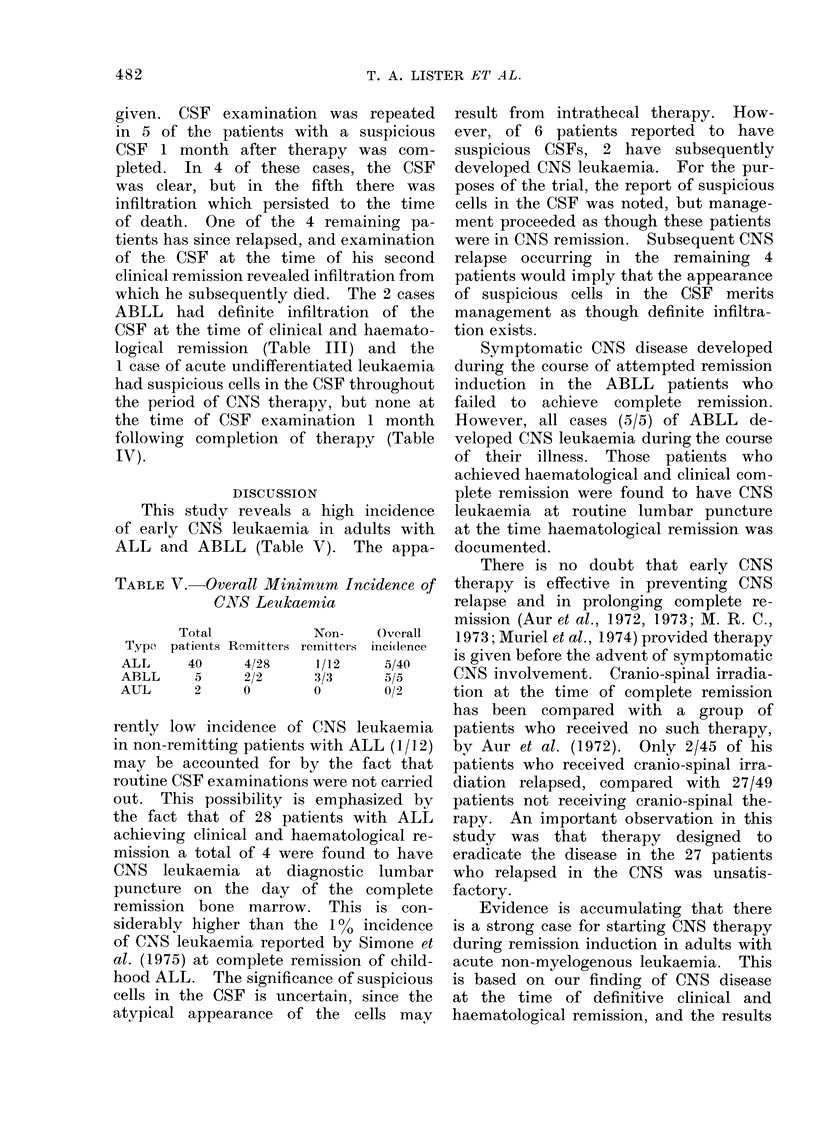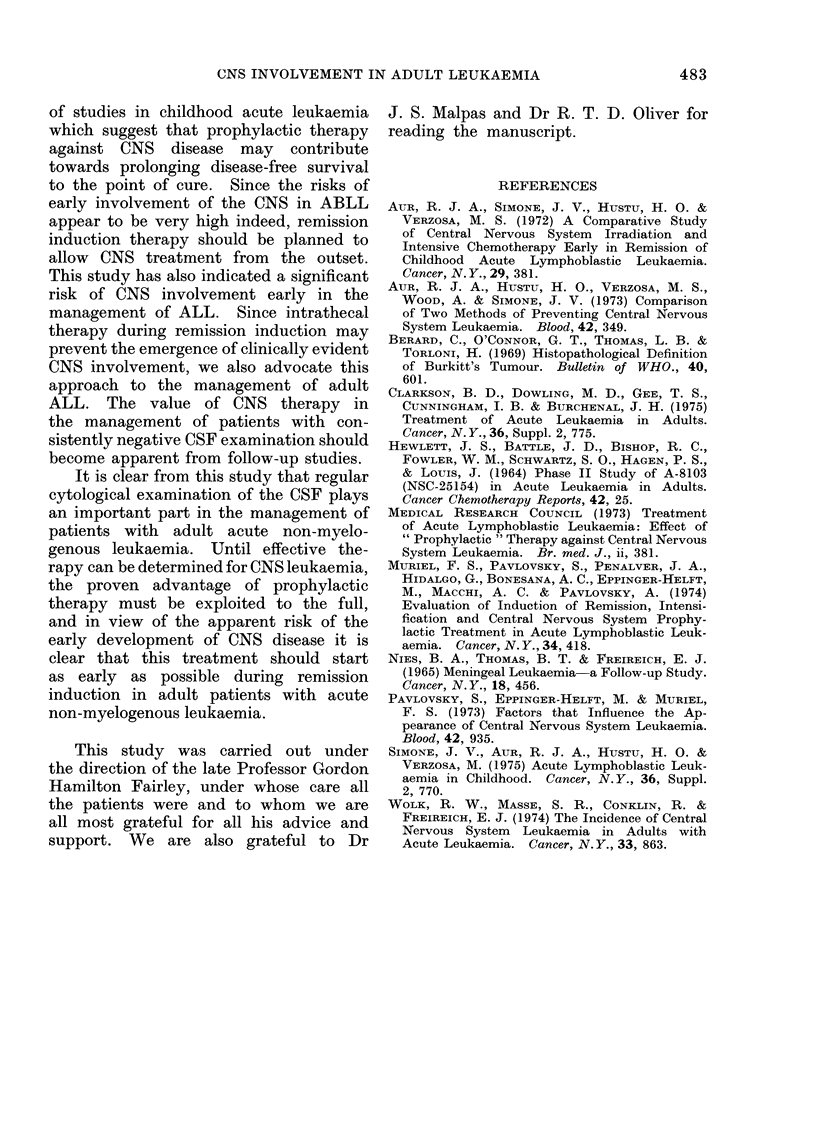# Early central nervous system involvement in adults with acute non-myelogenous leukaemia.

**DOI:** 10.1038/bjc.1977.71

**Published:** 1977-04

**Authors:** T. A. Lister, J. M. Whitehouse, M. E. Beard, A. Paxton, R. L. Brearley, L. Brown, P. F. Wrigley, D. Crowther

## Abstract

Of 47 consecutive patients aged 15-60 years with acute non-myelogenous leukaemia (ANML) (40 acute lymphoblastic leukaemia (ALL); 5 acute Burkitt-like leukaemia (ABLL), 2 acute undifferentiated leukaemia (AUL) treated with a standard chemotherapy protocol (OPAL), 31 achieved complete remission (28/40(70%) of patients with ALL). CNS leukaemia occurred in 4/16 non-remitters, and in 6 patients who achieved complete remission (CR). CNS leukaemia occurred in all 5 patients with acute Burkitt-like leukaemia. 4/28 patients with ALL achieving CR had evidence of CSF involvement on cytocentrifuge examination shortly after CR. The apparent risk of early CNS disease suggests that prophylactic CNS therapy should be given early in the treatment of acute non-myelogenous leukaemia.


*Br. J. Cancer* (1977) **35**, 479

### EARLY CENTRAL NERVOUS SYSTEM INVOLVEMENT IN ADULTS WITH ACUTE NON-MYELOGENOUS LEUKAEMIA

T. A. LISTER, J. M. A. WHITEHOUSE*, M. E. J. BEARD, A. PAXTON, R. L. BREARLEY, L. BROWN, P. F. M. WRIGLEY AND D. CROWTHER†

*From the I.C.R.F. Department of Medical Oncology and the Department of Haematology, St Bartholomew's Hospital, London EC1A 7BE*
**CRC Professor of Medical Oncology, Southampton General Hospital, Southampton SO9 4XY*
*† CRC Professor of Medical Oncology, Manchester University, and Christie Hospital, Manchester M20 9BX*

Received 18 March 1976 Accepted 25 November 1976

**Summary.—Of 47 consecutive patients aged 15–60 years with acute non-myelogenous leukaemia (ANML) (40 acute lymphoblastic leukaemia (ALL); 5 acute Burkitt-like leukaemia (ABLL), 2 acute undifferentiated leukaemia (AUL)) treated with a standard chemotherapy protocol (OPAL), 31 achieved complete remission (28/40 (70%) of patients with ALL). CNS leukaemia occurred in 4/16 non-remitters, and in 6 patients who achieved complete remission (CR). CNS leukaemia occurred in all 5 patients with acute Burkitt-like leukaemia. 4/28 patients with ALL achieving CR had evidence of CSF involvement on cytocentrifuge examination shortly after CR. The apparent risk of early CNS disease suggests that prophylactic CNS therapy should be given early in the treatment of acute non-myelogenous leukaemia.**

EFFECTIVE TREATMENT has altered the natural history of many malignancies. This is well illustrated by the emergence of central nervous system (CNS) disease during prolonged remission of children with acute lymphoblastic leukaemia (ALL). Children developing leukaemic infiltration of the CNS relapse systemically earlier than those who do not. Prophylactic therapy designed to prevent such CNS relapse has led to a significant improvement in survival and to possible cures (Aur *et al.*, 1972).

The results of treatment of adult ALL are less satisfactory than those achieved for childhood acute leukaemia, but nonetheless improvements in survival have also been achieved (Clarkson *et al.*, 1975). On the basis of childhood studies, it seems likely that prophylactic CNS treatment will contribute to improved survival in adult patients achieving complete remission. The incidence of CNS involvement in adult ALL has received less attention than that in childhood. Analysis is complicated by the fact that some studies include both children and adults. Nies, Thomas and Freireich (1965) compared the incidence of meningeal leukaemia in two series of patients with ALL, the first diagnosed between 1953 and 1958 and the second between 1961 and 1963. The incidence of symptomatic CNS disease was the same in both groups, despite improved survivals in the latter study. However, the overall incidence of CNS involvement was 25% in the first group and 42% in the second. The difference was accounted for by the detection of asymptomatic disease by cerebro-spinal fluid (CSF) examination in the second study. A real increase in the incidence of CNS involvement in association with prolonged survival is suggested by Pavlovsky, Eppinger-Helft and Muriel (1973) and Wolk *et al.* (1974).

Reprint requests to Dr. T. A. Lister, I.C.R.F. Department of Medical Oncology, St Bartholomew's Hospital, London EC1A 7BE.

Neither of these groups routinely examined the CSF of asymptomatic patients, but both used similar criteria for the recognition of involvement, namely the finding of leukaemic blast cells in the CSF. In Pavlovsky's study, the incidence of CNS involvement appeared to increase to 19% at 20 months.

The finding by Simone *et al.* (1975) that 1% of children have leukaemic blast cells in the CSF, either at presentation or at the time of early CNS therapy, suggests that a detailed study of patterns of CNS disease in adult ALL may be of value in determining the optimum management which is required to prevent CNS infiltration. A study of CNS disease in patients with acute non-myelogenous leukaemia between 15 and 60 years of age has been carried out at St Bartholomew's Hospital. This paper reports the results of a 3-year study.

### MATERIALS AND METHODS

Forty-seven consecutive adult patients aged between 15 and 60 years with acute non-myelogenous leukaemia (40 ALL; 5 acute Burkitt-like leukaemia (ABLL); 2 acute undifferentiated leukaemia (AUL)) admitted to St Bartholomew's Hospital between November 1972 and June 1976 were treated with a standard protocol (OPAL). The diagnosis was established by examination of peripheral blood and bone marrow aspirate smears. Acute non-myelogenous leukaemia was diagnosed when the frequency of blasts in the bone marrow exceeded 30% and when these showed no evidence of myeloid differentiation. Three categories of acute non-myelogenous leukaemia were recognized; ALL was identified on the basis of infiltration with blast cells of lymphoid appearance on Romanowsky staining, which were Sudan Black negative and some of which showed " block positive " staining with the Periodic Acid–Schiff (PAS) reaction. ABLL was recognized when the infiltrating blastic cells had deeply basophilic vacuolated cytoplasm on the Romanowsky stain (Berard *et al.*, 1969). These were all negative with the PAS reaction. A final category included those cases in which the infiltrating cells were not morphologically lymphoid or myeloid, and negative with both Sudan Black and PAS (AUL).

*OPAL protocol.*—The combination of vincristine and prednisolone, although highly effective in childhood ALL, is less effective in the adult variants. In consequence, Erwinia asparaginase (Porton) (10,000 u/m$^2$) and prednisolone (40 mg) were given daily for the first 14 days, and adriamycin (30 mg/m$^2$) was given in a fast-running i.v. infusion of normal saline plus vincristine (2 mg) at weekly intervals. Injections of vincristine and adriamycin were postponed if there was cytopenia associated with marrow hypoplasia. The marrow was assessed 1 week after the fourth injection of vincristine and adriamycin and, if clinical and haematological remission had not been achieved, a further 2 injections were given at weekly intervals and the marrow reassessed 1 week after the second. For a diagnosis of complete remission there had to be clinical, haematological and CNS freedom from disease. The criteria laid down by Hewlett *et al.* (1964) for peripheral blood and bone marrow remission were used. Remission was not recognized, however, if abnormal blasts could be recognized in the bone marrow, even if they constituted less than 5% of the total nucleated count. All patients achieving complete remission received early CNS therapy, consisting of cranial irradiation of 2400 rad (over 3 weeks) and intrathecal methotrexate (12·5 mg twice weekly for 5 doses).

*CNS leukaemia.*—A cytocentrifuge preparation of the CSF was examined in all patients achieving clinical and haematological remission, and a further 5 examinations were made during intrathecal therapy. The CSF of patients failing to achieve clinical and haematological remission was only studied if there was a clinical indication. CSF examination was carried out within 1 h of lumbar puncture; the smears were made using the cytocentrifuge (Shandon). Aliquots (0·5 ml) of CSF were centrifuged in duplicate at 1000 rev/min for 5 min. The smears were dried and then stained by May–Grünwald–Giemsa. Cytochemical stains were also employed if appropriate, but in practice these were not usually helpful in deciding whether there was leukaemic infiltration. Following examination of the stained preparations, samples were categorized into " negative ", " infiltrated " or

" infiltration suspected ". Infiltration was diagnosed when there were at least 5 leukaemic blast cells present in the cytocentrifuged deposit. The number 5 was chosen on an empirical basis. Samples were classified as negative when no leukaemic blast cells could be identified, even when the total white count was otherwise raised. Infiltration was suspected when an occasional blast cell could be identified, but the total of blast cells seen in the deposit was <5. The appearance of cells in the deposit with a high nuclear/cytoplasmic ratio and slightly immature chromatin, frequently with irregular and scanty cytoplasm, was also regarded as suspicious of infiltration. Following treatment, CNS remission was accepted when 2 consecutive CSF deposits were free from blast cells.

### RESULTS

Forty-seven cases were treated using the OPAL protocol and there were 31 complete remissions (details in Table I).

TABLE I.—*Results of Remission Induction in Acute Non-myelogenous Leukaemia*

| Diagnosis | Total | Remission | Non-remission |
|---|---|---|---|
| ALL | 40 | 28 (70%) | 12 |
| ABLL* | 5 | 2 | 3 |
| AUL† | 2 | 1 | 1 |

* Acute Burkitt-like leukaemia.
† Acute undifferentiated leukaemia.

*CNS leukaemia in patients failing to achieve clinical or haematological remission*

TABLE II.—*CNS Leukaemia in Non-remitters*

| Diagnosis | Total | Clinical disease | CSF disease |
|---|---|---|---|
| ALL | 12 | 1 | 0 |
| ABLL | 3 | 3 | 3 |
| AUL | 1 | 0 | 0 |

Sixteen patients receiving the OPAL protocol failed to achieve complete remission. One of the 12 cases of ALL developed focal epilepsy in the presence of retinal infiltrate. The platelet count was normal and the epileptiform convulsions were presumed to result from leukaemic infiltration. All 3 cases of ABLL failing to achieve clinical and haematological remission developed cranial nerve palsies. CSF examination revealed blast cells, but in one of these cases the CSF specimen was contaminated with blood in which there were circulating blast cells. Neither of the 2 cases of AUL had clinical symptoms or signs to suggest CNS infiltration, and routine CSF examination was not carried out.

*CNS leukaemia in patient achieving clinical and haematological remission* (Tables III and IV)

There were 31 patients in this group. Four out of 28 patients with ALL were found to have definite blast cells in the CSF, either on the first or on subsequent CSF examinations during early CNS therapy (Table III). A further 6 patients with ALL had suspicious cells in the CSF during the same period (Table IV). Since the significance of the suspicious cells was uncertain, and might have been related to intrathecal methotrexate, no further specific therapy was

TABLE III.—*CNS Leukaemia in Patients with Clinical and Haematological Remission*

| Type | Total | CSF$^-$ | CSF$^+$ | CSF$^\pm$ |
|---|---|---|---|---|
| ALL | 28 | 18 | 4 | 6 |
| ABLL | 2 | 0 | 2 | 0 |
| AUL | 1 | 0 | 0 | 1 |

TABLE IV.—*Follow-up on Patients with Suspicious Cells in the CSF at Clinical and Haematological Remission*

| Diagnosis | CSF 1 month after end of CNS therapy | Further follow-up |
|---|---|---|
| 1. ALL | Not done | Lost |
| 2. ALL | − | Continuing CR for 2 months |
| 3. ALL | − | Continuing CR for 4 months |
| 4. ALL | − | Continuing CR for 15 months |
| 5. ALL | − | + at 2nd haematological remission |
| 6. ALL | + | Frequent CNS relapses |
| 7. AUL | − | Continuing CR for 21 months |

given. CSF examination was repeated in 5 of the patients with a suspicious CSF 1 month after therapy was completed. In 4 of these cases, the CSF was clear, but in the fifth there was infiltration which persisted to the time of death. One of the 4 remaining patients has since relapsed, and examination of the CSF at the time of his second clinical remission revealed infiltration from which he subsequently died. The 2 cases ABLL had definite infiltration of the CSF at the time of clinical and haematological remission (Table III) and the 1 case of acute undifferentiated leukaemia had suspicious cells in the CSF throughout the period of CNS therapy, but none at the time of CSF examination 1 month following completion of therapy (Table IV).

### DISCUSSION

This study reveals a high incidence of early CNS leukaemia in adults with ALL and ABLL (Table V). The apparently low incidence of CNS leukaemia in non-remitting patients with ALL (1/12) may be accounted for by the fact that routine CSF examinations were not carried out. This possibility is emphasized by the fact that of 28 patients with ALL achieving clinical and haematological remission a total of 4 were found to have CNS leukaemia at diagnostic lumbar puncture on the day of the complete remission bone marrow. This is considerably higher than the 1% incidence of CNS leukaemia reported by Simone *et al.* (1975) at complete remission of childhood ALL. The significance of suspicious cells in the CSF is uncertain, since the atypical appearance of the cells may result from intrathecal therapy. However, of 6 patients reported to have suspicious CSFs, 2 have subsequently developed CNS leukaemia. For the purposes of the trial, the report of suspicious cells in the CSF was noted, but management proceeded as though these patients were in CNS remission. Subsequent CNS relapse occurring in the remaining 4 patients would imply that the appearance of suspicious cells in the CSF merits management as though definite infiltration exists.

TABLE V.—*Overall Minimum Incidence of CNS Leukaemia*

| Type | Total patients | Remitters | Non-remitters | Overall incidence |
|---|---|---|---|---|
| ALL | 40 | 4/28 | 1/12 | 5/40 |
| ABLL | 5 | 2/2 | 3/3 | 5/5 |
| AUL | 2 | 0 | 0 | 0/2 |

Symptomatic CNS disease developed during the course of attempted remission induction in the ABLL patients who failed to achieve complete remission. However, all cases (5/5) of ABLL developed CNS leukaemia during the course of their illness. Those patients who achieved haematological and clinical complete remission were found to have CNS leukaemia at routine lumbar puncture at the time haematological remission was documented.

There is no doubt that early CNS therapy is effective in preventing CNS relapse and in prolonging complete remission (Aur *et al.*, 1972, 1973; M. R. C., 1973; Muriel *et al.*, 1974) provided therapy is given before the advent of symptomatic CNS involvement. Cranio-spinal irradiation at the time of complete remission has been compared with a group of patients who received no such therapy, by Aur *et al.* (1972). Only 2/45 of his patients who received cranio-spinal irradiation relapsed, compared with 27/49 patients not receiving cranio-spinal therapy. An important observation in this study was that therapy designed to eradicate the disease in the 27 patients who relapsed in the CNS was unsatisfactory.

Evidence is accumulating that there is a strong case for starting CNS therapy during remission induction in adults with acute non-myelogenous leukaemia. This is based on our finding of CNS disease at the time of definitive clinical and haematological remission, and the results of studies in childhood acute leukaemia which suggest that prophylactic therapy against CNS disease may contribute towards prolonging disease-free survival to the point of cure. Since the risks of early involvement of the CNS in ABLL appear to be very high indeed, remission induction therapy should be planned to allow CNS treatment from the outset. This study has also indicated a significant risk of CNS involvement early in the management of ALL. Since intrathecal therapy during remission induction may prevent the emergence of clinically evident CNS involvement, we also advocate this approach to the management of adult ALL. The value of CNS therapy in the management of patients with consistently negative CSF examination should become apparent from follow-up studies.

It is clear from this study that regular cytological examination of the CSF plays an important part in the management of patients with adult acute non-myelogenous leukaemia. Until effective therapy can be determined for CNS leukaemia, the proven advantage of prophylactic therapy must be exploited to the full, and in view of the apparent risk of the early development of CNS disease it is clear that this treatment should start as early as possible during remission induction in adult patients with acute non-myelogenous leukaemia.

This study was carried out under the direction of the late Professor Gordon Hamilton Fairley, under whose care all the patients were and to whom we are all most grateful for all his advice and support. We are also grateful to Dr J. S. Malpas and Dr R. T. D. Oliver for reading the manuscript.

### REFERENCES

AUR, R. J. A., SIMONE, J. V., HUSTU, H. O. & VERZOSA, M. S. (1972) A Comparative Study of Central Nervous System Irradiation and Intensive Chemotherapy Early in Remission of Childhood Acute Lymphoblastic Leukaemia. *Cancer, N.Y.*, **29**, 381.

AUR, R. J. A., HUSTU, H. O., VERZOSA, M. S., WOOD, A. & SIMONE, J. V. (1973) Comparison of Two Methods of Preventing Central Nervous System Leukaemia. *Blood*, **42**, 349.

BERARD, C., O'CONNOR, G. T., THOMAS, L. B. & TORLONI, H. (1969) Histopathological Definition of Burkitt's Tumour. *Bulletin of WHO.*, **40**, 601.

CLARKSON, B. D., DOWLING, M. D., GEE, T. S., CUNNINGHAM, I. B. & BURCHENAL, J. H. (1975) Treatment of Acute Leukaemia in Adults. *Cancer, N.Y.*, **36**, Suppl. 2, 775.

HEWLETT, J. S., BATTLE, J. D., BISHOP, R. C., FOWLER, W. M., SCHWARTZ, S. O., HAGEN, P. S., & LOUIS, J. (1964) Phase II Study of A-8103 (NSC-25154) in Acute Leukaemia in Adults. *Cancer Chemotherapy Reports*, **42**, 25.

MEDICAL RESEARCH COUNCIL (1973) Treatment of Acute Lymphoblastic Leukaemia: Effect of " Prophylactic " Therapy against Central Nervous System Leukaemia. *Br. med. J.*, ii, 381.

MURIEL, F. S., PAVLOVSKY, S., PENALVER, J. A., HIDALGO, G., BONESANA, A. C., EPPINGER-HELFT, M., MACCHI, A. C. & PAVLOVSKY, A. (1974) Evaluation of Induction of Remission, Intensification and Central Nervous System Prophylactic Treatment in Acute Lymphoblastic Leukaemia. *Cancer, N.Y.*, **34**, 418.

NIES, B. A., THOMAS, B. T. & FREIREICH, E. J. (1965) Meningeal Leukaemia—a Follow-up Study. *Cancer, N.Y.*, **18**, 456.

PAVLOVSKY, S., EPPINGER-HELFT, M. & MURIEL, F. S. (1973) Factors that Influence the Appearance of Central Nervous System Leukaemia. *Blood*, **42**, 935.

SIMONE, J. V., AUR, R. J. A., HUSTU, H. O. & VERZOSA, M. (1975) Acute Lymphoblastic Leukaemia in Childhood. *Cancer, N.Y.*, **36**, Suppl. 2, 770.

WOLK, R. W., MASSE, S. R., CONKLIN, R. & FREIREICH, E. J. (1974) The Incidence of Central Nervous System Leukaemia in Adults with Acute Leukaemia. *Cancer, N.Y.*, **33**, 863.